# How to Conduct Store Observations of Tobacco Marketing and Products

**DOI:** 10.5888/pcd13.150504

**Published:** 2016-02-18

**Authors:** Ashley L. Feld, Trent O. Johnson, Katherine W. Byerly, Kurt M. Ribisl

**Affiliations:** Author Affiliations: Ashley L. Feld, Katherine W. Byerly, Gillings School of Global Public Health, University of North Carolina, Chapel Hill, North Carolina; Trent O. Johnson, Stanford Prevention Research Center, Stanford University School of Medicine, Palo Alto, California.

## Abstract

As tobacco companies continue to heavily market their products at the point of sale, tobacco control groups seek strategies to combat the negative effects of this marketing. Store observations, which have been widely used by researchers and practitioners alike, are an excellent surveillance tool. This article provides a guide for public health practitioners interested in working in the tobacco retail environment by detailing the steps involved in conducting store observations of tobacco marketing and products including 1) obtaining tobacco product retailer lists, 2) creating measures, 3) selecting a mode of data collection, 4) training data collectors, and 5) analyzing data. We also highlight issues that may arise while in the field and provide information on disseminating results of store observations, including the potential policy implications.

## Introduction

In recent years, tobacco control groups have become increasingly interested in the point of sale (POS), or the stores in which tobacco products are sold. A 2014 survey of tobacco control staff indicated that most states are conducting POS store observations at the local level (1). The tobacco industry spends the overwhelming majority of its marketing and promotional budget at the POS for both cigarettes (92.1%) and smokeless tobacco (71.3%); most of this spending is dedicated to price discounts (eg, sale price, coupons) (2,3). Collectively, tobacco companies spend close to $1 million per hour at the POS (2,3). Researchers have examined the effects of exposure to POS marketing and have determined that it prompts tobacco cravings (4) and unplanned purchases (5), undermines quit attempts (6,7), and leads to increased initiation of tobacco use (8–10).

Tobacco control researchers have emphasized that surveillance is a key first step to understanding how to combat the negative effects of POS marketing (11,12). Observing tobacco stores is imperative to understanding the retail environment, informing appropriate tobacco control interventions for individual communities, and evaluating interventions, including policy change. A systematic review by Lee and colleagues indicated that published research articles on tobacco store observations increased from about 1 per year in the early 1990s to nearly 10 per year since 2010 (13). Although this review (13) did not detail how to conduct store observations, it noted that store observation data are generally reliable. The purpose of this article is to provide public health practitioners with an overview of the process of conducting tobacco store observations for advocacy or evaluation efforts. Specifically, we outline sources for tobacco store lists, measures and modes of data collection, training and field support issues, tips for data analysis, and potential dissemination and policy strategies.

## Tobacco Store Lists

Before you begin your store observations, define the goals of your inquiry and the geographic area of interest. Store observation projects range from single neighborhoods to cities or even entire states. The 4 most common ways to compile tobacco store lists are 1) obtaining licensing or enforcement lists, 2) using Synar reports for identifying other data sources, 3) purchasing business lists, and 4) using “ground truthing” or “canvassing” (ie, conducting a manual in-person survey of all streets in the target geographic area).

Most US states require stores to have a license to sell tobacco, and some communities also maintain their own licensing lists (14). If a license is required to sell tobacco in your state or community, a licensing list (often publicly available from the licensing agency) offers the best place to start.

Another source of store lists is each state’s publicly available Synar report (15).Although these annual reports required by the federal government focus on compliance with youth access laws, they also contain information on the sampling strategy used to identify stores (eg, this report [16] describes California’s approach), which may help you find a source to create your own list. Most Synar reports can be found online (15). Licensing lists for alcohol or liquor are an indirect source that can help identify tobacco retailers, because many stores selling alcohol also sell tobacco products.

Business lists, which classify stores by type, are also available for purchase from companies such as ReferenceUSA and Dun and Bradstreet. Although business lists cannot tell you which stores sell tobacco products, you can select store types that typically sell tobacco products (tobacco stores, supermarkets and grocery stores, convenience stores, gas stations with convenience stores, other gas stations, warehouse clubs and supercenters, news dealers and newsstands, liquor stores, pharmacies, discount department stores) (17).

Finally, if you are working in a small geographic area and have ample staff or volunteers, you may be able to “ground truth” or “canvas” your community by walking or driving primary and secondary roads and documenting tobacco stores (17,18). A 3-county study comparing business lists and ground truthing found that purchased lists identified most tobacco stores (ReferenceUSA identified 82%, and Dun and Bradstreet identified 69%; combined, they identified 90%) but also included many “false positives” that should be removed (17). However, ground truthing can be expensive and time consuming. More detailed information on choosing a store list is provided (Table 1).

**Table 1:** Pros and Cons of Store List Sources as the Sampling Frame for Store Observations at Tobacco Retail Outlets

Pros/Cons	Tobacco Licensing Lists	Business Lists	Ground Truthing	State Synar Reports
**Pros**	Fairly accurate list that will not require extensive cleaning and verification	Available for the whole United States and can be purchased for a certain geographic area (eg, by zip code, county, state) and by store type	The only way to be sure you have a list of all tobacco retailers in your area of interest	May point you to an alternate source for obtaining a store list
	All stores licensed to sell tobacco	Updated regularly	Can identify sample and conduct audit simultaneously	
	Often publicly available and free or inexpensive			
**Cons**	Not all jurisdictions require licensing	Cannot limit exclusively to tobacco retailers, only store types likely to sell tobacco	Time-intensive and costly	Methods used for identifying stores vary widely; lists may not include all tobacco retailers
	May not be updated regularly			
	Often contain address and telephone errors	Must complete thorough list cleaning and tobacco sale verification		
	Agencies may not be able to share them	Price is typically per store record and can be expensive		

Short of ground truthing, you should find an alternative way to clean your list to confirm which stores sell tobacco products. Depending on the quality of the list, there are many cleaning steps you should consider: 1) removing stores outside your geographic area of interest; 2) removing duplicate or invalid addresses, or both; and 3) removing store chains known not to sell tobacco (eg, Target, CVS). Business lists typically require more thorough cleaning than do licensing lists. Once you have completed the initial list cleaning, we recommend calling all stores in your sample to confirm whether they sell tobacco products, which saves time and effort in the field.

You will need to carefully select your sample of stores for observations. Given the importance and complexities involved with sample selection, we recommend consulting a statistician or epidemiologist to discuss the pros and cons of various sampling methods and to ensure you have an adequate sample size to meet your analysis goals. If your focal area is small, you may be able to complete an observation of all tobacco stores (a census). In larger geographic areas, some type of random sampling from your store list will be more efficient and increase the likelihood that your sample is representative of your geographic area. Other advanced multistage sampling strategies exist; for example, you can randomly sample counties, zip codes, or census tracts and then sample stores in these areas. If your resources are limited or you have a specific goal in mind, you may consider a more strategic sampling strategy such as stores within 1,000 feet of schools or stores in areas with certain sociodemographic characteristics (eg, neighborhoods with a higher percentage of residents living in poverty). Sociodemographic data for your community or state can be found online at www.factfinder.census.gov or www.city-data.com.

## Measures

Data can be collected on varied measures related to the retail environment, so be sure to use your campaign goals to inform your selections. For instance, if you are concerned about youth and adult use of menthol tobacco products, ensure that you collect data on menthol-flavored products, price promotions, and prices of leading brands (eg, Newport). Table 2 outlines measures that have been used in POS observations and connects them with example policy implications.

**Table 2:** Examples of Store Observation Measures for Tobacco Retail Outlets and Subsequent Potential Policy Implications

Measure	Description and Examples	Potential Policy Implications
**Store characteristics**		
Location	Store address and latitude/longitude	Licensing or zoning policies can be used to limit the density and number of tobacco retailers by imposing minimum distance requirements between existing tobacco retailers, capping the number of tobacco retailers (in a geographic area or proportional to population size), or prohibiting tobacco sales in specific venues (eg, pharmacies) or within a certain distance of youth-populated areas (eg, no tobacco retailers within 1,000 ft of schools)
Store type	Tobacco stores, supermarkets and grocery stores, convenience stores, gas stations with convenience stores, other gas stations, warehouse clubs and supercenters, news dealers and newsstands, liquor stores, pharmacies, discount department stores, dollar stores	
Pharmacy counter	Captures whether a store has a pharmacy counter, regardless of store type	
Proximity of tobacco retailers to other locations	Measure of proximity (eg, feet or blocks) and locations (eg, near schools or parks)	
**Marketing materials**		
Branded signs	Branded materials include the brand insignia, imagery, font, and/or colors (often advertise a specific product and/or a price discount)	Marketing materials can be addressed by policies that restrict the time (eg, requiring stores to conceal marketing materials during K-12 school hours) place (eg, no marketing materials within 3 ft of candy, ice cream, or soda fountains), and manner (eg, restricting size of branded displays) of advertising
Branded displays	Portable units that merchandise tobacco products	
Branded shelving units	Usually floor-to-ceiling in height and located behind the counter, used to merchandise products, and have clearly branded header at the top	
Branded functional items	Branded items that serve another functional purpose in addition to advertising the product (eg, change mat, gas pump topper)	
**Product availability and packaging**		
Product categories	Cigarettes, cigars, cigarillos, little cigars, smokeless (snus, chew, moist/dry snuff, dip), electronic smoking products (e-cigarettes, e-hookah, e-cigars, e-liquid, e-cartridge), roll-your-own tobacco, pipe tobacco, hookah	Examples of restrictions on product availability and packaging include restricting sale of flavored products, restricting sale of menthol cigarettes, or implementing a minimum pack size for cigars and cigarillos
Product qualities	Flavored, menthol, pack size, etc	
**Product placement**		
Self-service of products	Ability for customers to access products without clerk assistance	Examples include restrictions on self-service of cigars, cigarillos, or e-cigarettes
Youth appeal	Products below 3 feet; products within 12 inches of candy or other items that appeal to youth	
**Price promotions**		
Special price discounts	Offer a limited-time reduced price (eg, “special offer,” “cents-off”)	An example is a ban on coupon redemption or other price promotions
Multi-pack discounts	Offer an incentive for a customer to buy more than 1 item (eg, “only $xx when you buy 2 packs”)	
Buy some, get some	Provide an additional item for free or at a discount with purchase (eg, “buy 2 packs, get 1 free” or “free snus with the purchase of a pack”)	
**Prices**		
Advertised or cashier provided price	Cheapest or advertised price for a specific product, brand, and variety/pack size; price data can be analyzed with or without sales tax (it can be time-consuming to calculate removing taxes after data collection)	The most common price restriction policy is a minimum price law
Advertised price of cheapest product		
Prices under $1		
**Counter-marketing**		
Point of sale health warnings (graphic or text) or cessation information	Counter-advertising mechanisms that use written messages and/or pictorial (or “graphic”) warnings of the health impacts of smoking; may be on marketing materials or tobacco packaging or as stand-alone signs	Policy implications can include requirements or voluntary agreements for retailers to display health warning signs and/or cessation information
**Compliance**		
Measures that evaluate whether stores are in compliance with existing laws on sales and marketing of tobacco products	Some enforcement agencies already collect this data	May reveal need for policy enforcement

The easiest way to select measures is by adopting an existing store observation instrument. Recently, a group of researchers and practitioners under the National Cancer Institute (NCI)–funded State and Community Tobacco Control Initiative released a set of recommended store observation measures and training materials called STARS (Standardized Tobacco Assessment for Retail Settings) (19). Using standard measures allows for greater comparability between states and communities and is more time-efficient than creating measures from scratch. If you choose to modify STARS or create your own measures, remember that it is better to fully address a few key topic areas than to attempt to collect more data than you have resources to analyze and report.

If creating your own store observation questions, closed-ended answer choices are easier to answer and analyze than open-ended questions. Some store observations count the number of marketing materials present at the store (eg, number of cigarette signs). However, counting is time-consuming and has few related policy solutions. In many cases, measuring the presence of an item (yes or no) is enough, or you may use stratified categories (eg, 0, 1–3, 4–5, 6 or more). You can also design measures to capture specific brands, product categories, or product qualities. For example, you can ask questions about Marlboro price promotions, signs for e-cigarettes, or availability of flavored products (Table 2).

Data are traditionally collected about both the exterior and the interior of the store, although most of the advertising and all of the products appear inside. Exterior measures (eg, price promotions on building exterior, fuel pumps, or fences) help to gauge potential youth and adult exposure to tobacco marketing and promotions that occurs as people pass by or enter a store. Interior measures often focus on the area around the checkout counter but should include the entire store. When deciding whether to collect interior or exterior data first, think about which variables are most important for your goals. Remember that you are more likely to experience a refusal on the interior of the store, leaving you with only exterior data in those stores. If exterior-only data are less relevant to your group, then consider collecting interior data first.

After your survey development is complete, you may need approval by the ethics review board in your organization. However, most store observations are exempt from human subjects requirements because they are public observations and rarely ask questions about identifiable people.

## Mode of Data Collection

Data can be recorded in several ways, each with different advantages concerning content, time, quality, and cost. Although most store observations have used paper-and-pencil instruments in the past, new technologies such as electronic data collection have been used successfully (18,20,21). Other innovative methods are also being investigated including crowd-sourcing (22) and capturing data with cameras and coding the images post–data collection (23).

When selecting a mode of data collection, consider the following: complexity of questions; time required to train, collect, and transcribe data; data quality; and cost (Table 3). For example, although paper surveys are low cost, they require significant staff time to enter data after collection. Electronic data collection using devices such as smartphones may have higher startup costs (for devices and programming), but they can save time on data entry, improve data quality, and are less conspicuous.

**Table 3:** Pros and Cons of Data Collection Modes for Store Observations at Tobacco Retail Outlets

Pros/Cons	Paper Data Collection	Smart Phones and Tablets
**Types of questions**	No constraints on type of questions	Questions limited to types included in survey software
	No skip logic to program but easy to skip questions on paper	Skip patterns and data validation built into many programs
		Smartphones reduce types of questions possible due to screen size
**Cost**	Lower startup cost but significant personnel cost to enter data	Possible high startup cost for devices if not using existing devices or devices of volunteers
	Inexpensive	Software and programming cost
**Time**	No need to train on how to use device	Must train on how to use device
	Significant time required for data entry; potential for data entry errors	Significant time saved on data entry
		Often more time to program and test electronic survey than to create a paper/pencil survey
**Other pros**	NA	Inconspicuous
		Usually includes advanced features such as built-in camera and GPS (global positioning system)
		Ability to monitor data as it is uploaded
		Fewer data entry errors (eg, skip logic, response validation)
**Other cons**	Conspicuous	May require technical expertise to program
	Less ability to identify and correct data entry errors as they occur	Possibility of theft or damage to device
	Inability to measure exact location without additional GPS device	

Abbreviation: NA, not applicable.

## Data Collection, Training, and Field Support

All store observations, large and small, require training and field support to ensure that data collectors can accurately and safely conduct store observations. You should always pilot test before beginning full data collection in at least 5 to 10 stores in varied store types and neighborhoods. This process will ensure operability, assess timing and length, and indicate other contextual factors that may be unique to the setting or to the data collectors. Leave time to revise your survey and training materials based on what you learn.

Store observation data collectors range from seasoned public health veterans or professional surveyors to volunteer youth. Data collectors can be recruited from many sources including community groups, schools, colleges, or community coalitions. Paid data collectors can also be sourced from contract research organizations or temporary agencies. When possible, use data collectors familiar with the neighborhood and cultural norms of the community. If working with youth, you may need to make accommodations, such as obtaining parental consent, assigning chaperones, and organizing transportation. Moreover, your data collection will be confined to establishments that allow youth access (ie, no liquor stores, bars, or tobacco stores). Merchants may also respond differently to underage youth than they would to adults and could be more or less willing to assist them in collecting price data for products they are legally unable to purchase. Two advantages of working with youth are that they are typically less intimidating to store owners and managers, and they are compelling speakers during the dissemination and policy advocacy phase. All groups are capable of collecting valid and reliable data, but the design of the survey, training, and support should be tailored to the skills of your data collectors.

We strongly encourage trainings to be conducted in person and to include a visual training presentation and a printed manual. Include logistic details such as how to use store lists, obtaining transportation to and between stores, how to enter a store, whether and how to introduce oneself, and taking safety precautions. We also recommend quizzing data collectors on key concepts and conducting simulated store observations at nearby stores. You may consider sending 2 data collectors to a small sample of the same stores so that you can examine interrater reliability.

Before beginning any training, try to anticipate the questions and needs of your data collectors. Here are some common concerns of data collectors:

**Store cooperation.** During pilot testing, note how cooperative store employees are with your data collectors, and determine the best strategy to use in your community. Create a mock script that describes the project:

Hello, my name is _______. I am working [with _______] on a project looking at different consumer products that are sold in stores. I’m going to look around for a few minutes, but I will not get in the way of your customers. Please know that I’m not an inspector or working for another retailer. Thank you for your help.

You may include information about whether you are specifically assessing tobacco products or if your survey assesses tobacco, food, and alcohol. If questioned, you should be clear about how data will be reported (in aggregate form or not) and whether store identities will be kept anonymous. In addition, an official letter from your organization about the data collection (on letterhead) can be a useful tool to show the legitimacy of the activity.

An important goal of training is to instruct data collectors to be respectful of the store environment and to avoid interrupting the normal flow of business. Although merchant interaction can often be avoided in larger stores, it becomes necessary when prompted by a merchant or if survey questions require information that is not apparent (eg, price is not displayed). Some store observations are conducted with full transparency and with scheduled store visits. Other surveys attempt to collect data without interacting with merchants and supplying a letter of explanation only when prompted. Our research teams have been successful in using a hybrid approach in which data collectors are overt only in small stores. Of the 36 studies included in the review by Lee et al of tobacco store observations (13), 42% collected data covertly, and 39% notified store staff as part of their protocol. Do what is appropriate in your community and comfortable for your organization; however, it is important to train data collectors on how to enter the store and interact with merchants. This interaction with merchants can be tested and experimented with during the piloting phase.

**Safety.** Safety is a top priority and can be increased by grouping data collectors in pairs and ensuring access to a mobile phone. Many groups conducting store observations with youth require they be accompanied by a trusted adult. Every data collector should have the opportunity to decline a store observation in a location that makes him or her feel unsafe.

Staff should be on call during all times that store observations may occur to provide assistance with any questions or issues that arise in the field. At a minimum, data collectors should be supplied with a support telephone number and prescribed hours for data collection. If resources allow, staff should catalog questions from the field and monitor collected data for quality assurance. Any updates or clarifications should be communicated to data collectors in a timely manner.

## Data Analysis and Dissemination

Once you have collected store observation data, it is important to clean the data of mistakes and format them in a usable way. For example, ensure that your price data are consistent — either with or without sales tax. After cleaning the data, you should analyze them to find patterns in your community, especially in relationship to demographic data (eg, pattern shows more tobacco marketing materials in low-income neighborhoods). If you do not have a statistician or epidemiologist on staff, you may be able to consult one from a partner organization that is familiar with the retail environment (eg, nutrition, alcohol).

In addition to writing reports, maps are a powerful and accessible way to visualize the data you have collected. Although software systems (eg, geographic information system [GIS]) are useful for making maps, you can also use Google Maps to create a map for free (visit www.maps.google.com, sign into a free Google account, use the menu to click on “My Maps,” and then “Create Map”). Maps are an easily understandable way to tell the story of what is happening in your community.

Data reports and maps can be used when interacting with policy makers or creating informational campaigns. For example, if you were educating policy makers about the health impact of tobacco products near schools, you could bring a map to your city council meeting that shows the tobacco stores and amount of tobacco marketing near the schools in your community — especially of flavored and inexpensive products that appeal to youth. Maps can also be included in press releases to local media outlets as a visual aid to help the public understand the problem.

We also recommend that you refer to www.CounterTobacco.org for more dissemination resources, including maps and news stories of recent POS activities happening around the country. Many communities are active in POS policy change (24), and groups can often connect and learn from one another’s experiences using store observation data to inform public opinion and local policy. You could also consider partnering with nutrition or alcohol groups that may also be interested in observing the retail environment. Doing this may not only help increase resources (eg, data collectors, statisticians), but it is also a great exercise in coalition building.

In sum, store observations are a useful tool to monitor tobacco products at the POS. State and local organizations may use store observation data to raise public awareness about the sales and marketing of tobacco products and to inform policy changes (see Table 2) (13). Importantly, these types of policies can help reduce youth tobacco use (25), counteract disparities in tobacco industry targeting (26), and should be part of a comprehensive tobacco control program (27,28).
